# Supplementary material for: Multi-method proof-of-concept evaluation for R2Play: a novel multi-domain return-to-play assessment tool for concussion
Source: PLOS Digit Health. 2025 Oct 14;4(10):e0001049. doi: 10.1371/journal.pdig.0001049 (PMC12520354; doi:10.1371/journal.pdig.0001049)
Supplement: S2 Appendix — Caption: S2 Appendix provides the interview guides used for this work. The youth interview guide appears first, followed by the clinician post-assessment interview guide, and then the clinician follow-up interview guide. (PDF) [file pdig.0001049.s002.pdf]

S2 Appendix: Interview guides

Youth Interview Guide

The purpose of the interview is to see what you thought about our assessment. The questions are open, and you can bring up anything you think is important. The interview will be audio recorded. You do not have to answer any question you feel uncomfortable with, and you can stop the interview at any time. Your responses will be confidential, which means we won't use your name in any findings we publish. We might use some quotes from the interviews may be used, but we will not say your name. The interview will be audio recorded so that the research team can keep a record of the interview. This will let the team review it and analyze the responses along with other participants' responses to help with our research study. Do you have any questions for me?

**Focus area**	**Example questions**
General impression	1. What was your favorite part of the assessment?<br>2. What was your least favorite part of the assessment?<br>3. What do you think about our idea?<br>a) Was it too hard? Too easy?<br>b) Was it fun?<br>4. What did you think about how long the assessment took?<br>a) Did it feel too long or too short?
Skills in multi-domain assessment	5. Did this remind of you the skills you do in sports?<br>6. Did you have a hard time learning the 'rules'?
Feedback and performance	7. Do you think the levels were in a good order or sequence? If you could choose, would you have done them in a different way?<br>8. Can you think of any helpful information we could give you during the assessment?<br>a) Do you want to know how you're doing?<br>9. Can you think of any information we could give you after the assessment that would be helpful?
Wearable sensors	10. Did you like wearing the sensors during the assessment?<br>a) Were they uncomfortable?<br>11. Did you like seeing your heart rate?

Clinician Post-Assessment Interview Guide

The purpose of this interview is a quick reflection on your experience with the *R2Play* assessment. We will be following up again with a phone call after your second session, so we can get a more in-depth understanding of what you think about the assessment. For now, we just want your first impressions! The interview will be audio recorded. You do not have to answer

any question you feel uncomfortable with, and you can stop the interview at any time. Your responses will be confidential and anonymized in any findings we publish. Quotes from the interviews may be used, but it will not be possible to identify their source. The interview will be audio recorded so that the research team can keep a record of the interview. This will let the team review it and analyze the responses along with other participants' responses to help with our research study. Do you have any questions for me?

**Focus area**	**Example questions**
Result interpretation	1. Do you think the assessment would be informative in RtoP decision making?<br>2. If this individual had been recently concussed, how would you use these results to inform an RtoP clearance decision?<br>3. Did you think the results were useful?<br>4. Would you like to have seen any other results?
System Refinement	5. Do you think the levels were in a good order or sequence? If you could choose, would you have done them in a different way?

Clinician Follow-Up Interview Guide

The purpose of this interview is to reflect on your experience with the *R2Play* assessment. The questions will be open-ended to allow you the chance to raise the issues that you feel are important. The interview will be audio recorded. You do not have to answer any question you feel uncomfortable with, and you can stop the interview at any time. Your responses will be confidential and anonymized in any findings we publish. Quotes from the interviews may be used, but it will not be possible to identify their source. The interview will be audio recorded so that the research team can keep a record of the interview. This will let the team review it and analyze the responses along with other participants' responses to help with our research study. Do you have any questions for me?

**Focus area**	**Example comments and questions**
Acceptability	6. What do you think about our idea for the *R2Play* assessment?<br>7. What did you like about it? What didn't you like about it?
Demand	8. Would you consider using this assessment in practice?<br>a) Do you think this is an informative assessment?<br>b) Do you think this assessment is an improvement in return-to-play?<br>c) Do you think this is a fun assessment for youth athletes?<br>d) Do you think this assessment emulates skills that are important in sports?<br>9. Did you feel like you had enough control over the assessment? Would have liked more control? (e.g. was assessment too prescribed)

	10. Did it take away from your interaction with the athlete?
Implementation/ Practicality	11. What would make this easier for you to implement in practice? (e.g. cost, space, time).<br>a) Which of these do you think will be the biggest challenges?<br>12. How long do you think the assessment should be?<br>a) Were there any parts you felt were not useful/could be removed?
Expansion	13. Would you have liked to see anything else in the assessment overall?<br>14. How do you think we could improve on the assessment?
Adaption	15. Do you think this assessment could be useful for different populations? For example, younger kids, non-athletes, or adult athletes?
