# Supplementary material for: Multi-method proof-of-concept evaluation for R2Play: a novel multi-domain return-to-play assessment tool for concussion
Source: PLOS Digit Health. 2025 Oct 14;4(10):e0001049. doi: 10.1371/journal.pdig.0001049 (PMC12520354; doi:10.1371/journal.pdig.0001049)
Supplement: S3 Appendix — presents a design change table that was constructed to summarize participant suggestions in relation to the design objectives, and map feedback onto subsequent design iterations. Design changes suggestions from clinicians are presented first, followed by design change suggestions from youth. (PDF) [file pdig.0001049.s003.pdf]

S3 Appendix: Design change table

CLINICIANS			
**Domain**	**Code**	**Description**	**Compatibility and design changes**<br>Implemented / Iterating / Incompatible
Easy to use	Age-appropriate symptom scale	Using developmentally appropriate symptom scales for younger children instead of the adolescent PCSI	Currently, one uniform symptom scale is required across all participants to enable consistent analyses. However, an option to select from multiple symptom scales could be considered in the future.
	Writing notes during breaks	Allowing clinicians to add observation notes during breaks	Enabled notes during breaks.
	Scramble example in level training	Showing an example of the scramble condition during level training	Added animated example of Scramble condition to level training screen.
	Colour-coded heart rate	Using colour-coded system to display participant heart rates based on exercise intensity ranges	Added colour-coded system to display participants' heart rate in exercise intensity ranges using percentage of their age-predicted maximal heart rate.
	Editing notes	Allowing clinicians to edit previous observation notes in case of wrong selections	Enabled editing for observation notes.
	Assessment protocol handout	Providing clinicians with handouts or resources summarizing assessment protocol and level instructions	Implemented assessment "map" within clinician interface, with current assessment step highlighted, and developed training resources depicting overall assessment flow and individual level instructions.
	Starting breaks automatically	Starting breaks automatically once participants finish the repetition, rather than starting breaks manually	Programmed breaks to start automatically once the participant finishes the repetition
	Heart rate rules for breaks	Adding rules or guidelines for when to start the next level based on participant heart rate	Developed preliminary guidelines for when to offer extended breaks based on participants' heart rate, to be used in future studies. Will consider adding rules into the interface in the future.

Fun	Feedback on time to increase competitiveness	Showing participants their time/score between levels as a form of motivation for competitive athletes	The implications of time/score feedback on assessment scoring are unclear at this time. Feedback between levels will be considered in future versions of *R2Play*.
Sport-like	Verbal direction	Requiring participants to actively follow verbal directions rather than simply ignoring irrelevant background noises	The effects of the current background noise condition are not fully understood. Verbal direction could be considered in future versions of *R2Play*.
	High and low tablets	Adjusting the height of each tablet so that some tablets require bending down and others reaching up to better resemble sport	Personalizing the height of tablet targets would complicate the set-up process. This could be reconsidered in future versions of *R2Play* once current system set up is deemed manageable.
	Incorporating virtual reality (VR) to increase immersivity	Using VR to increase immersivity of *R2Play.*	We chose not to use VR in *R2Play* due to confounding symptoms between VR sickness and concussion. The static nature of VR is also not compatible with *R2Play* aim for dynamic assessment.
	Lengthening assessment to fatigue athletes	Making the assessment longer to fully fatigue athletes and test their cognitive abilities after prolonged exercise	Longer assessment conflicts with the design objective of resource efficiency.
	Insufficient exertion	Some clinicians felt that *R2Play* was not physically demanding enough and should require greater physical exertion.	Standardized the exercise level task (burpees) to ensure adequate exertion. Will continue to monitor the physical exertion requirements of the task and make further adjustments as needed.
	Sport-specific skills	Having participants use sport-specific skills or equipment (e.g., holding a racquet or stick handling) while completing *R2Play*	*R2Play* is intended to reflect general skills required across many sports. Sport-specific customizations could be considered in future versions.
Potentially clinically valuable	Symptom check-in after each repetition	Repeating the symptom check-in after every repetition rather than only between levels	More check-ins would substantially increase assessment duration, which conflicts with the design objective of resource efficiency. Frequent symptom monitoring during exercise testing may also have iatrogenic effects.

	Standardization	Standardizing the exercise task and whether participants are warned of repetition conditions	Standardized the exercise task for future studies (burpees) and added specific instructions in clinician training not to warn participants of conditions.
	Concussion rehabilitation tool	Potential to use *R2Play* as a rehabilitation tool to address fear avoidance and encourage more exercise.	Work currently underway to purposefully adapt *R2Play* as an active rehabilitation therapy tool.
Resource efficient	Removing Go-No-Go level	Removing Go-No-Go level due to overlap with Stroop level	Removed Go-No-Go level to streamline assessment.
	Shortening to two repetitions per level	Shortening each level to two repetitions instead of four	The value of each repetition condition is unclear and requires further evaluation. The second standard condition in each level could be removed in the future.
	Set-up	Making sure that clinicians can quickly and easily set up the *R2Play* system for an assessment session	Streamlined equipment set-up process and developed set-up guides as part of *R2Play* clinician training resources.
	Time	Time as potential barrier to clinical implementation of *R2Play*. Reducing overall duration to enable integration in practice.	Removed Go-No-Go level to streamline assessment and programmed rest breaks to start automatically. Will continue to monitor assessment duration and make further changes as needed.
	Training	The need for clinicians to have training on how to understand and interpret *R2Play* assessment results	*R2Play* clinician training program currently under development. Training resources will be evaluated and iteratively refined in future studies.
Flexible	Control over rest breaks	Having control over the duration of rest breaks between repetitions based on participants' condition	Reduced the duration of breaks from 30 to 20 seconds and added option for clinicians to add another 10 seconds to breaks as needed.
	Flexibility with levels	Ability to change order of levels, repeat levels, or end assessment early due to participants' condition and/or technical issues.	For psychometric validation, assessment delivery must be standardized. Future adaptation as a rehabilitation tool could enable more flexibility with levels.
	Younger children	Simplifying *R2Play* for use with younger children (e.g., reducing number of characters in	Work currently underway to purposefully adapt *R2Play* for younger children.

		trail, using symbols instead of numbers and letters)			
**YOUTH**					
**Domain**	**Code**	**Description**	**Compatibility and design changes**		
			Implemented	Iterating	Incompatible
Fun	Creating competitive aspect	Creating a "leaderboard" for participants to see how their performance compares to other people	*R2Play* is still undergoing iterative refinement, and more data is needed to make normative comparisons. Norms and leaderboards will be considered in future versions of *R2Play*.		
Sport-like	Spacing out more	Spreading the tablet targets over a larger space to allow longer strides and more running	Larger space requirement could be a barrier to clinical implementation and conflicts with the design objectives of resource efficiency and flexibility.		
	Sport-specific skills	Including sport-specific skills or equipment (e.g., running between bases) while completing *R2Play*	*R2Play* is intended to reflect general skills required across many sports. Sport-specific customizations could be considered in future versions.		
Potentially clinically valuable	Heart rate zones	Showing the exercise intensity level that participants reach based on their heart rate "zones"	Added colour-coded system to display participants' heart rate in exercise intensity ranges using percentage of their age-predicted maximal heart rate.		
	Increasing exercise repetitions	Increasing the number of exercise task repetitions in the exercise level to increase exertion levels	Standardized the exercise level task (burpees) to ensure adequate exertion. Will continue to monitor the physical exertion requirements of the task and make further adjustments as needed.		
	Adding more scrambles	Having more than one scramble occur within the scramble condition	The effects of the current scramble condition are not fully understood. A second scramble cue could be considered in future versions of *R2Play*.		
