# Supplementary material for: Multi-method proof-of-concept evaluation for R2Play: a novel multi-domain return-to-play assessment tool for concussion
Source: PLOS Digit Health. 2025 Oct 14;4(10):e0001049. doi: 10.1371/journal.pdig.0001049 (PMC12520354; doi:10.1371/journal.pdig.0001049)
Supplement: S5 Appendix — presents the raw RPE results from every level for each youth participant. (PDF) [file pdig.0001049.s005.pdf]

S5 Appendix: RPE results

Participant	Motor Trail (Pre)	Number-Letter Level	Exercise Level	Go-No-Go Level	Stroop Level	Motor Trail (Post)
Y1	1	4	5	4	5	3
Y2	0	2	4	4	4	4
Y3	1	3	6	6	7	7
Y4		1	2	2	2	2
Y5	1	2	2	2	3	3
Y6	1	2	2	1	1	1
Y7	1	2	4	3	5	3
Y8	1	3	3	4	4	4
Y9	0	1	1	1	2	0
Y10	0	1	4	5	6	6
