# Supplementary material for: Multi-method proof-of-concept evaluation for R2Play: a novel multi-domain return-to-play assessment tool for concussion
Source: PLOS Digit Health. 2025 Oct 14;4(10):e0001049. doi: 10.1371/journal.pdig.0001049 (PMC12520354; doi:10.1371/journal.pdig.0001049)
Supplement: S6 Appendix — provides the baseline PCSI scores for each youth participant, as well as their symptom check-in response for each level. (PDF) [file pdig.0001049.s006.pdf]

S6 Appendix: Baseline PCSI scores and symptom check-in responses

**Participant**	**Baseline PCSI Score**	**Symptom Check-In Score***					
		**Motor Trail (Pre)**	**Number-Letter Trail**	**Exercise Trail**	**Go-No-Go Trail**	**Stroop Trail**	**Motor Trail (Post)**
Y1	11	1	1	1	1	1	1
Y2	2	0	0	0	0	0	0
Y3	0	0	0	0	0	0	0
Y4	0	0	0	0	0	0	0
Y5	5	0	0	0	0	0	0
Y6	7	0	0	0	0	0	0
Y7	6	0	0	0	0	0	0
Y8	0	0	0	0	0	0	0
Y9	5	0	0	0	0	0	0
Y10	3	0	0	0	0	0	0
Median (IQR)	4 (6)	0 (0)	0 (0)	0 (0)	0 (0)	0 (0)	0 (0)

*0 = No symptoms, 1 = Some symptoms but did not get worse, 2 = Symptoms got worse, 3 = Symptoms so much worse I had to stop.
