# Supplementary material for: Multi-method proof-of-concept evaluation for R2Play: a novel multi-domain return-to-play assessment tool for concussion
Source: PLOS Digit Health. 2025 Oct 14;4(10):e0001049. doi: 10.1371/journal.pdig.0001049 (PMC12520354; doi:10.1371/journal.pdig.0001049)
Supplement: S7 Appendix — presents the raw R2Play error results from every level for each youth participant. (PDF) [file pdig.0001049.s007.pdf]

S7 Appendix: *R2Play* error results

Participant	Number Letter Level			Exercise Level			Go-No-Go Level			Stroop Level			Total
	Std	Aud	Scr	Std	Aud	Scr	Std	Aud	Scr	Std	Aud	Scr	
Y1	0	0	2	1	1	1	0	0	2	0	0	0	7
Y2	0	0	3	1	1	1	0	0	0	0	0	0	6
Y3	0	0	4	1	1	1	0	1	0	0	0	4	12
Y4	0	0	0	1	1	1	0	0	0	0	0	0	3
Y5	0	0	0	1	7	1	0	0	0	2	0	0	11
Y6	0	0	0	1	1	6	0	0	0	0	0	0	8
Y7	0	0	2	1	1	1	0	0	1	0	0	0	6
Y8	0	1	7	1	1	2	0	0	1	0	0	0	13
Y9	-	-	-	-	-	-	-	-	-	-	-	-	-
Y10	0	0	1	1	1	2	0	0	1	0	0	0	6
**Total**	0	1	19	9	15	16	0	1	5	2	0	4	72
	20			40			6			6			

Std, Standard condition; Aud, Auditory condition; Scr, Scramble condition. *R2Play* performance data not available for Y9 due to a technical issue in system output.
