# Supplementary material for: Multi-method proof-of-concept evaluation for R2Play: a novel multi-domain return-to-play assessment tool for concussion
Source: PLOS Digit Health. 2025 Oct 14;4(10):e0001049. doi: 10.1371/journal.pdig.0001049 (PMC12520354; doi:10.1371/journal.pdig.0001049)
Supplement: S9 Appendix — outlines the R2Play system equipment and costs. Equipment components are labeled on an image of the system and described further in a table below, including their purpose and cost. (PDF) [file pdig.0001049.s009.pdf]

S9 Appendix: *R2Play* equipment and associated costs

Equipment items are identified by number in the image and described further in the table below

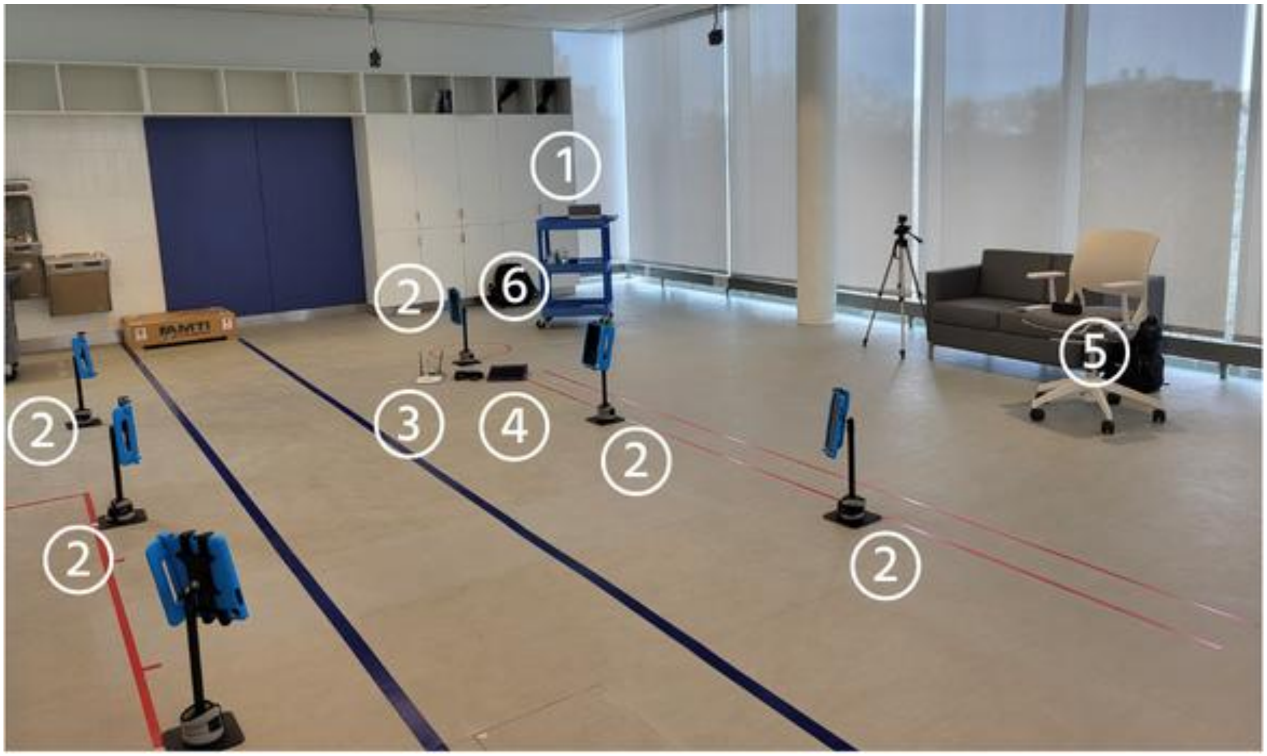

Number	Item	Purpose	Cost (CAD)
1	Clinician laptop (2-in-1 convertible Microsoft Surface Pro laptop)	Runs the *R2Play* clinician interface software used to control the tablets, administer the assessment, monitor clinical measures, and track performance	$1,300.00
2	Tablet buttons (6): tablet, case, stand, and weight for stability	Display the numbers and letters that participants interact with to connect the alphanumeric trail	$2,517.81
3	Wi-Fi router	Supplies local Wi-Fi network to connect tablets with clinician interface	$50.00
4	Polar H10 heart rate monitor and chest strap	Collects participant's heart rate and relays it to the clinician interface	$163.98
5	Bluetooth speaker	Plays assessment audio (start and stop noises, correct and incorrect selection cues, exercise and scramble tones)	$70.00
6	Utility cart	Used to transport equipment and serves as mobile workstation.	$193.00
		**Total**	$4,244.79
