# Supplementary material for: Multi-method proof-of-concept evaluation for R2Play: a novel multi-domain return-to-play assessment tool for concussion
Source: PLOS Digit Health. 2025 Oct 14;4(10):e0001049. doi: 10.1371/journal.pdig.0001049 (PMC12520354; doi:10.1371/journal.pdig.0001049)
Supplement: Attachment — Submitted filename: Response to Reviewers - R2Play Proof of Concept.docx [file pdig.0001049.s011.docx]

**Reviewer: 1**

This paper presents a proof-of-concept evaluation of R2Play, a novel multi-domain assessment tool designed to support return-to-play decision-making for youth with concussion. The authors used a multi-method approach to evaluate whether the R2Play prototype aligned with six design objectives: being easy to use, fun, sport-like, clinically valuable, resource efficient, and flexible. Five clinicians and ten healthy youth (ages 10-22) participated in testing sessions followed by semi-structured interviews. Quantitative measures included the System Usability Scale, heart rate monitoring, ratings of perceived exertion, completion times, errors, and multi-task cost scores. Results showed that R2Play demonstrated good-to-excellent usability (SUS=81±8.4), achieved moderate-to-high intensity exertion (peak HR=80±11% age-predicted maximal), and was perceived as engaging and sport-like by participants. Clinicians valued R2Play's potential for observing multi-domain skill integration relevant to return-to-play readiness, though they noted concerns about resource requirements and result interpretation. The authors documented iterative design improvements based on study findings, including streamlining the assessment by removing redundant levels, standardizing physical exercises, and enhancing the user interface. This study supports proof-of-concept for R2Play as a promising assessment tool while acknowledging the need for further validation with youth with concussion.

We thank the reviewer for their detailed review our manuscript.

**Reviewer: 2**

Thank you for the opportunity to review this thoughtful and well-executed study titled "Multi-method proof-of-concept evaluation for R2Play: a novel multi-domain return-to-play assessment tool for concussion." The manuscript addresses a clear and timely need in pediatric and youth concussion assessment, particularly in bridging the gap between clinical testing and sport-specific demands. Overall, the manuscript is clearly written and methodologically sound. The mixed-methods approach is appropriate for a proof-of-concept study and offers valuable insights into both system usability and the potential clinical value of R2Play. The qualitative and quantitative integration is effective and provides a comprehensive evaluation of the prototype. Attached is detailed comment aimed at enhancing clarity, accessibility, and utility for both research and clinical audiences.

We thank the reviewer for taking the time to review our manuscript and for their kind words.

**Reviewer Comments to the Authors**

Dear Authors,

Thank you for the opportunity to review this innovative and thoughtfully conducted study on R2Play. The manuscript is well-written and addresses a clear gap in concussion management, especially in paediatric and youth sports contexts. Below are my detailed comments aimed at strengthening the manuscript and improving clarity and impact.

**1. Title and Abstract**

- **Line 1–3:** The title is informative but could be shortened for impact. Consider removing “proof-of-concept” and focusing on “multi-method evaluation” since the abstract already elaborates on the pilot nature.
  Thank you for this suggestion. We feel that the “proof-of-concept” label in the title is important to delineate this distinct phase of work within the broader context of the *R2Play* research program, which also includes a subsequent feasibility study (recently submitted for publication) and cross-site pilot study (currently underway).
- **Lines 24–45:** The abstract is generally strong. However, phrases like “sport-like” and “multi-domain” are used frequently. Consider rephrasing one of them for variation. Also, explain briefly what “multi-task cost scores” represent for readers unfamiliar with the term.
  Thank you for highlighting this. We have added a brief explanation of “multi-task cost scores” within the abstract (line 34) and edited the author summary (line 55) for readers who may not be familiar with the concept of multi-task cost scores.

**2. Introduction**

- **Lines 67–90:** Excellent contextualization of the problem. The authors make a compelling case for the necessity of multi-domain assessments in RtoP decision-making.
  Thank you for your kind words and positive feedback.
- **Line 85:** “dynamic changing environment” could be reworded as “rapidly evolving environment” for better flow.
  Thank you for this wording suggestion. We have implemented the edits as described.
- **Lines 94–105:** The description of the R2Play system is engaging. Consider clarifying earlier what “alphanumeric trail” entails before introducing the Trail Making Test example.
  Thank you for your kind words and suggestion. We have moved the “(1-A-2-B-3-C)” clarification to be right after mention of “alphanumerical order” and before the Trail Making Test example.

**3. Materials and Methods**

- **Line 117:** The multi-method approach is well-justified. However, a brief rationale for choosing paired testing (clinicians with youth) would strengthen the design justification.
  We appreciate this feedback and suggestion. We have added a sentence to describe the rationale for paired testing with clinicians and youth (line 121).
- **Lines 144–150:** The rationale for excluding recently concussed youth is logical, but this limitation should be stated explicitly in the discussion later.
  Thank you for raising this issue. We agree that this is an important consideration and therefore acknowledge this issue in the limitations section of the discussion (line 724).
- **Lines 223–239:** The explanation of multi-domain cost score calculations is comprehensive. Still, some readers may benefit from a visual example of the score derivation in the supplementary appendix.
  Thank you for highlighting this important issue of explaining the multi-domain cost scores in ways that are meaningful and easily understandable for diverse audiences. As noted in the future directions section (line 730), this is important future work, and we are actively exploring the best way to do this through consultation with our knowledge user collaborators (youth advisors and clinician partners). We hope to include co-created visual examples in future publications and knowledge mobilization materials.

**4. Results**

- **Lines 311–318:** The authors have done well to manage data completeness issues. It may help to clarify which technical issues most frequently occurred.
  We thank the reviewer for their kind words and understanding regarding technical issues and data completeness issues in this preliminary proof-of-concept study. Fortunate, each technical issue only occurred once and was subsequently rectified to prevent re-occurrence. We have clarified this in text (line 328).
- **Line 329:** The SUS score interpretation is useful, but a visual summary in a table or figure would enhance accessibility.

We appreciate this suggestion to enhance accessibility. Given the length of this manuscript and the number of other tables and figures presented, we have opted to present only key aggregate SUS data within the manuscript text itself. Per PLOS policies, we have included the raw SUS data in supplementary material (S4 Appendix) for readers who wish to see this data visualised in table format.

- **Lines 408–420:** Table 4 is helpful, though consider shading or bolding peak values to guide the reader's eye more easily.
  Thank you for this suggestion. We have bolded key information in Table 4 to better catch the reader’s attention.
- **Lines 499–507:** Completion time costs are clearly higher in cognitive tasks. This is a key finding and could be emphasized more directly in the results narrative.
  Thank you for highlighting this important finding. We have added further clarification in the results narrative (lines 508-511) and further emphasized this finding and its implications for *R2Play* in the discussion (line 693-696).

**5. Discussion**

- **Line 526–567:** The section on clinical value is particularly strong. The inclusion of rich quotes improves the narrative. However, consider expanding on how the system may support rehabilitation beyond assessment.
  Thank you for these kind words. We agree that findings regarding clinical value are particularly promising and have added a sentence in the discussion (line 720) to highlight participant feedback regarding how the system may support rehabilitation beyond assessment.
- **Line 594–615:** The discussion of time and cost constraints is honest and relevant. Including a cost-effectiveness comparison to existing assessments would strengthen the argument for clinical feasibility.
  This is an important point, and we appreciate the reviewer’s positive feedback. While cost-effectiveness analysis is beyond the scope of this initial proof-of-concept study, we do address the cost issue more directly in our subsequent feasibility manuscript (currently under review). We have added an acknowledgement of this important issue in the future directions section (lines 731-732).

**6. General Style and Language**

- Avoid overuse of colons and dashes. For example:
  - Line 74: Replace colon with “such as”
  - Line 376: Reword “tediousness and stress” to “potential monotony or stress triggers”

Thank you for this feedback. We have made minor stylistic and language changes throughout the manuscript in line with the reviewer’s comments.

- Phrases such as “easy to use, fun, sport-like” (e.g., Line 28) appear often and begin to lose impact. Consider substituting synonyms or rephrasing.
  We appreciate this wording suggestion. Since these terms refer to the specific design objectives that the paper aims to address (as described in Table 1), synonyms or paraphrasing may be confusing for the reader as to which objective we are referring to throughout the paper.

**7. Figures and Tables**

- **Figure 1 and 3**: These are clear and aid understanding. However, they could benefit from more descriptive captions, particularly for readers who may not read the full text.
  Thank you for this suggestion. We have added more details to the Figure 3 caption as suggested.
- **Tables**: Well formatted. However, in Table 2, adding a small sample value in the formula example would be helpful for unfamiliar readers.
  We thank the reviewer for this suggestion. We have added an additional column in Table 2 to provide an example of the calculation and resulting value for each cost score measure.

**Summary**

This is an impressive and much-needed study introducing a novel tool to enhance clinical return-to-play decision-making. The writing is polished, and the methodology is rigorous for a proof-of-concept design. With minor refinements to terminology repetition, deeper justification for design decisions, and enhanced visual summaries, the manuscript will be a strong candidate for publication.

Recommendation: **Minor Revisions**
