# Supplementary material for: Multi-method proof-of-concept evaluation for R2Play: a novel multi-domain return-to-play assessment tool for concussion
Source: PLOS Digit Health. 2025 Oct 14;4(10):e0001049. doi: 10.1371/journal.pdig.0001049 (PMC12520354; doi:10.1371/journal.pdig.0001049)
Supplement: Attachment — Submitted filename: My review.docx [file pdig.0001049.s012.docx]

**Peer Review Comments to the Authors**

**General Assessment**

This is a timely and well-executed proof-of-concept study introducing R2Play, a novel, low-cost, multi-domain digital tool for assessing return-to-play (RtoP) readiness in youth post-concussion. The multi-method design involving paired clinician-youth testing and both quantitative and qualitative analyses is appropriate for the study's stage and aims. The manuscript demonstrates strong methodological planning, a clear articulation of design objectives, and a meaningful integration of participant feedback into tool refinement. It reflects a commendable example of user-centered development in digital health.

That said, there are a few areas where the manuscript could be improved to enhance accessibility, theoretical framing, interpretability, and utility for both clinical and research audiences.

**Overall Framing and Contribution**

1. **Clarify R2Play's comparative novelty:** While the system is clearly innovative, the manuscript could more clearly position R2Play relative to existing concussion tools or dual-task assessments, especially digital platforms. A comparative table in the introduction or discussion would help establish its distinctive value proposition.
2. **Tighter linkage to existing concussion guidelines:** The paper would benefit from explicitly referencing how R2Play could complement or enhance existing return-to-play frameworks (e.g., the Berlin Consensus Statement) or be integrated into current clinical protocols.

**Design Objectives and Conceptual Rationale**

1. **More explicit conceptual framework:** While the design objectives (ease of use, fun, sport-like, etc.) are commendable, the rationale behind selecting these particular criteria could be deepened. Are they rooted in prior user-centered design frameworks or implementation science principles? Making this clearer would lend more theoretical robustness.
2. **Consider re-grouping the objectives:** Several objectives overlap (e.g., “fun” and “sport-like” or “resource efficiency” and “flexibility”). Consider condensing into higher-level themes like ***“User Experience,” “Clinical Relevance,” and “Implementation Feasibility”*** to streamline the narrative and discussion.

**Sample and Generalizability**

1. **Small, homogeneous sample:** While typical for a proof-of-concept, the manuscript should more clearly acknowledge that the clinician sample is heavily skewed toward rehabilitation professionals, potentially limiting generalizability to broader clinical or sports medicine audiences.
2. **Youth sample characteristics:** It would be helpful to add more detail about the socioeconomic, racial/ethnic, or geographic diversity of the youth participants. These factors are often highly relevant in concussion incidence, care access, and engagement with digital tools.

**Quantitative Analyses**

1. **Better visual representation of cost scores:** While the cost score table is comprehensive, it’s quite dense and hard to interpret. Consider supplementing with a bar or line plot to show performance trade-offs across domains and levels.
2. **Missing data treatment:** The manuscript briefly mentions technical issues leading to missing HR and completion data, but the methods for handling this missingness are not described. Even if data were dropped listwise, this should be stated explicitly.
3. **Statistical inference or effect estimation**: While the authors correctly avoid over-quantification for a small pilot, even basic confidence intervals or variability estimates for key metrics (e.g., SUS scores, exertion levels) would improve scientific transparency.

**Qualitative Analysis**

1. **Greater integration with existing literature:** The qualitative findings (e.g., about gamification, cognitive load, or perceived clinical value) are rich but could be better contextualized by linking them to known challenges in concussion assessment or behavior change theory.
2. **Strengthen coding rigor:** The analysis section would benefit from more detail on how coding consistency was ensured. Was any inter-coder agreement calculated? Were member checks or triangulation methods employed?

**Implementation and Scalability**

1. **Hardware and software requirements:** Although R2Play is described as “low-cost,” no cost estimates are provided for the tablets, software development, or training. Adding even approximate ranges would help readers assess scalability.
2. **User training time:** A brief mention is made of training clinicians (~10 minutes), but further details would help illustrate feasibility across busy clinical environments. Would novice clinicians (e.g., medical residents or trainers) be equally comfortable?

**Discussion and Future Work**

1. **Deeper discussion of use in injured populations:** While future studies in concussed youth are acknowledged, the current data from healthy participants may not translate directly to clinical utility. Consider expanding the limitations section to explore this gap more explicitly.
2. **Longitudinal utility:** Could R2Play be used to track recovery over time? Or is it primarily designed for single-time-point decision-making? Clarifying this would help define its role in care pathways.
3. **Regulatory implications:** If R2Play is intended for broader clinical use, is there an intended regulatory path (e.g., as a medical device or digital health application)? This could be flagged in the future directions section.

**Writing and Presentation**

1. **Terminology:** The terms “multi-domain,” “multi-task,” and “sport-like” are somewhat overused. Consider occasional rephrasing (e.g., "ecologically valid," "real-world complexity") to maintain reader engagement.
2. **Figures and tables:** Captions could better explain what readers should take away. For example, Figure 4 could include brief interpretation notes, not just what is being displayed.
3. **Clarity in acronyms and metrics:** Ensure that all acronyms (e.g., RPE, PCSI) and metrics (e.g., “% of age-predicted HRmax”) are clearly defined the first time they appear, even in figures and appendices.

**Conclusion and Recommendation**

This manuscript makes a meaningful contribution to the evolving field of concussion assessment by creatively integrating digital technology, multi-domain tasks, and user-centered design. With minor revisions to clarify theoretical underpinnings, interpretability, and visual communication, the paper will be well-positioned for publication in PLOS Digital Health.
