# Supplementary material for: Multi-method proof-of-concept evaluation for R2Play: a novel multi-domain return-to-play assessment tool for concussion
Source: PLOS Digit Health. 2025 Oct 14;4(10):e0001049. doi: 10.1371/journal.pdig.0001049 (PMC12520354; doi:10.1371/journal.pdig.0001049)
Supplement: Attachment — Submitted filename: Response to Reviewers (Round 2) - R2Play Proof of Concept.docx [file pdig.0001049.s013.docx]

**Peer Review Comments to the Authors**

**General Assessment**

This is a timely and well-executed proof-of-concept study introducing R2Play, a novel, low-cost, multi-domain digital tool for assessing return-to-play (RtoP) readiness in youth post-concussion. The multi-method design involving paired clinician-youth testing and both quantitative and qualitative analyses is appropriate for the study's stage and aims. The manuscript demonstrates strong methodological planning, a clear articulation of design objectives, and a meaningful integration of participant feedback into tool refinement. It reflects a commendable example of user-centered development in digital health.

That said, there are a few areas where the manuscript could be improved to enhance accessibility, theoretical framing, interpretability, and utility for both clinical and research audiences.

We thank the reviewer for their kind words, detailed review, and thoughtful suggestions to help improve our manuscript.

**Overall Framing and Contribution**

1. **Clarify R2Play's comparative novelty:** While the system is clearly innovative, the manuscript could more clearly position R2Play relative to existing concussion tools or dual-task assessments, especially digital platforms. A comparative table in the introduction or discussion would help establish its distinctive value proposition.

Thank you for this helpful suggestion. We have added more detail to the introduction about existing dual-task assessments for concussion and prior digital approaches to dual-task assessment, including limitations of these approaches (lines 95-100). Considering the length of the manuscript and number of tables/figures, we opted not to include a comparison table, which we hope the reviewer understands. Readers seeking more information about existing dual-task assessments may wish to review our previous scoping review on existing multidomain concussion assessments, which is referenced in the manuscript (reference 23).

2. **Tighter linkage to existing concussion guidelines:** The paper would benefit from explicitly referencing how R2Play could complement or enhance existing return-to-play frameworks (e.g., the Berlin Consensus Statement) or be integrated into current clinical protocols.

Thank you for this feedback. We agree that this context would be helpful for the reader and have added more detail to the introduction and discussion regarding how *R2Play* could complement existing return-to-play frameworks (e.g., consensus statement) and clinical practices (lines 74-78 and 733-734).

**Design Objectives and Conceptual Rationale**

1. **More explicit conceptual framework:** While the design objectives (ease of use, fun, sport-like, etc.) are commendable, the rationale behind selecting these particular criteria could be deepened. Are they rooted in prior user-centered design frameworks or implementation science principles? Making this clearer would lend more theoretical robustness.

Thank you for raising this important issue. We have further clarified how these design objectives were selected and added a reference directing readers to our previous publication for more information about the specific rationale for each objective (lines 137-140).

2. **Consider re-grouping the objectives:** Several objectives overlap (e.g., “fun” and “sport-like” or “resource efficiency” and “flexibility”). Consider condensing into higher-level themes like ***“User Experience,” “Clinical Relevance,” and “Implementation Feasibility”*** to streamline the narrative and discussion.

Thank you for this useful suggestion. While we agree that there is overlap in some design objectives, we feel that grouping them in this way could detract attention from the specific objectives and confuse readers with additional sub-heading levels.

**Sample and Generalizability**

1. **Small, homogeneous sample:** While typical for a proof-of-concept, the manuscript should more clearly acknowledge that the clinician sample is heavily skewed toward rehabilitation professionals, potentially limiting generalizability to broader clinical or sports medicine audiences.

Thank you for highlighting this. We have now acknowledged the limited clinician sample in the limitations section (lines 743-747).

2. **Youth sample characteristics:** It would be helpful to add more detail about the socioeconomic, racial/ethnic, or geographic diversity of the youth participants. These factors are often highly relevant in concussion incidence, care access, and engagement with digital tools.

Thank you for raising this important issue of diversity and representation among our youth sample. Unfortunately, data on socioeconomic, racial/ethnic, and geographic factors are not available for this sample. We have thus added this consideration to the limitations section (lines 743-745). In subsequent studies we are now collecting this data, which we agree will be very important for understanding the psychometric properties of the *R2Play* assessment and engagement/adoption of this digital tool.

**Quantitative Analyses**

1. **Better visual representation of cost scores:** While the cost score table is comprehensive, it’s quite dense and hard to interpret. Consider supplementing with a bar or line plot to show performance trade-offs across domains and levels.

Thank you – we appreciate the importance of presenting the cost scores data in a way that is meaningful and easily understandable for diverse audiences. Through discussion with our team, we decided that a line plot (Figure 4) would be most appropriate at this stage to help visualize the underlying performance trade-offs across levels/conditions that make up the cost scores. As noted in the future directions section, finding ways to make the cost score results easier to interpret is important future work, and we are actively exploring the best way to do this through consultation with our knowledge user collaborators (youth advisors and clinician partners). We hope to include co-created visual examples in future publications and knowledge mobilization materials.

2. **Missing data treatment:** The manuscript briefly mentions technical issues leading to missing HR and completion data, but the methods for handling this missingness are not described. Even if data were dropped listwise, this should be stated explicitly.

Thank you for highlighting this. We have indicated how missing data was treated in the opening paragraph of the results section.

3. **Statistical inference or effect estimation**: While the authors correctly avoid over-quantification for a small pilot, even basic confidence intervals or variability estimates for key metrics (e.g., SUS scores, exertion levels) would improve scientific transparency.

Thank you for this important feedback and helpful suggestion to ensure that quantitative data is presented appropriately given the small sample size in this proof-of-concept study. We have added 95% confidence intervals for the SUS and exertion data as suggested.

**Qualitative Analysis**

1. **Greater integration with existing literature:** The qualitative findings (e.g., about gamification, cognitive load, or perceived clinical value) are rich but could be better contextualized by linking them to known challenges in concussion assessment or behavior change theory.

Thank you for this constructive feedback. We have edited the discussion to better link qualitative findings to current challenges in concussion management, existing concussion guidelines (per the reviewer’s other suggestion above), and gamification literature.

2. **Strengthen coding rigor:** The analysis section would benefit from more detail on how coding consistency was ensured. Was any inter-coder agreement calculated? Were member checks or triangulation methods employed?

Thank you for this feedback. We performed conventional content analysis as described by Hseih and Shannon (DOI: 10.1177/1049732305276687). Given the nature of this method and our philosophical perspective, quantification and calculation of inter-coder agreement did not align with our analytical approach. We have edited the analysis section to describe the strategies we used to support qualitative rigor and trustworthiness of our findings, including collaborative coding and consensus among multiple researchers.

**Implementation and Scalability**

1. **Hardware and software requirements:** Although R2Play is described as “low-cost,” no cost estimates are provided for the tablets, software development, or training. Adding even approximate ranges would help readers assess scalability.

Thank you – we agree that cost is an important consideration. Total cost of the *R2Play* system hardware is reported under the “system cost” section heading, and the cost of individual equipment components can be found in S9 Appendix. Software development costs are not reported as development work was completed prior to the present study, while the cost of user training will be considered in future work.

2. **User training time:** A brief mention is made of training clinicians (~10 minutes), but further details would help illustrate feasibility across busy clinical environments. Would novice clinicians (e.g., medical residents or trainers) be equally comfortable?

Thank you – we agree that clinician training is an important aspect of feasibility and have added further details to the protocol about how training was provided in this study. As noted in the future directions section, more comprehensive and standardized training materials are needed to support *R2Play* administration and interpretation. In subsequent work, we are now evaluating a clinician training program for *R2Play* across different clinical environments, which we have clarified in the future directions.

**Discussion and Future Work**

1. **Deeper discussion of use in injured populations:** While future studies in concussed youth are acknowledged, the current data from healthy participants may not translate directly to clinical utility. Consider expanding the limitations section to explore this gap more explicitly.

Thank you – we have added a stronger acknowledgement of this limitation (lines 740-741).

2. **Longitudinal utility:** Could R2Play be used to track recovery over time? Or is it primarily designed for single-time-point decision-making? Clarifying this would help define its role in care pathways.

Thank you for this suggestion. We have added to the discussion that R2Play may also be useful for tracking longitudinal recovery.

3. **Regulatory implications:** If R2Play is intended for broader clinical use, is there an intended regulatory path (e.g., as a medical device or digital health application)? This could be flagged in the future directions section.

Thank you for mentioning this important issue. We have added a sentence in the future directions regarding regulatory implications.

**Writing and Presentation**

1. **Terminology:** The terms “multi-domain,” “multi-task,” and “sport-like” are somewhat overused. Consider occasional rephrasing (e.g., "ecologically valid," "real-world complexity") to maintain reader engagement.

Thank you for this feedback. We have made stylistic and language changes throughout the manuscript in line with the reviewer’s comments.

2. **Figures and tables:** Captions could better explain what readers should take away. For example, Figure 4 could include brief interpretation notes, not just what is being displayed.

This is a great suggestion. We have edited the Figure 4 caption to explain more directly what readers should take away from the image.

3. **Clarity in acronyms and metrics:** Ensure that all acronyms (e.g., RPE, PCSI) and metrics (e.g., “% of age-predicted HRmax”) are clearly defined the first time they appear, even in figures and appendices.

Thank you. We have reviewed to ensure clarity and definition of all acronyms and metrics at first mention in the manuscript.

**Conclusion and Recommendation**

This manuscript makes a meaningful contribution to the evolving field of concussion assessment by creatively integrating digital technology, multi-domain tasks, and user-centered design. With minor revisions to clarify theoretical underpinnings, interpretability, and visual communication, the paper will be well-positioned for publication in PLOS Digital Health.
