# Supplementary material for: Multi-method proof-of-concept evaluation for R2Play: a novel multi-domain return-to-play assessment tool for concussion
Source: PLOS Digit Health. 2025 Oct 14;4(10):e0001049. doi: 10.1371/journal.pdig.0001049 (PMC12520354; doi:10.1371/journal.pdig.0001049)
Supplement: Attachment — Submitted filename: Reviewer Comments-PLOS-1.docx [file pdig.0001049.s010.docx]

**Reviewer Comments to the Authors**

Dear Authors,

Thank you for the opportunity to review this innovative and thoughtfully conducted study on R2Play. The manuscript is well-written and addresses a clear gap in concussion management, especially in paediatric and youth sports contexts. Below are my detailed comments aimed at strengthening the manuscript and improving clarity and impact.

**1. Title and Abstract**

- **Line 1–3:** The title is informative but could be shortened for impact. Consider removing “proof-of-concept” and focusing on “multi-method evaluation” since the abstract already elaborates on the pilot nature.
- **Lines 24–45:** The abstract is generally strong. However, phrases like “sport-like” and “multi-domain” are used frequently. Consider rephrasing one of them for variation. Also, explain briefly what “multi-task cost scores” represent for readers unfamiliar with the term.

**2. Introduction**

- **Lines 67–90:** Excellent contextualization of the problem. The authors make a compelling case for the necessity of multi-domain assessments in RtoP decision-making.
- **Line 85:** “dynamic changing environment” could be reworded as “rapidly evolving environment” for better flow.
- **Lines 94–105:** The description of the R2Play system is engaging. Consider clarifying earlier what “alphanumeric trail” entails before introducing the Trail Making Test example.

**3. Materials and Methods**

- **Line 117:** The multi-method approach is well-justified. However, a brief rationale for choosing paired testing (clinicians with youth) would strengthen the design justification.
- **Lines 144–150:** The rationale for excluding recently concussed youth is logical, but this limitation should be stated explicitly in the discussion later.
- **Lines 223–239:** The explanation of multi-domain cost score calculations is comprehensive. Still, some readers may benefit from a visual example of the score derivation in the supplementary appendix.

**4. Results**

- **Lines 311–318:** The authors have done well to manage data completeness issues. It may help to clarify which technical issues most frequently occurred.
- **Line 329:** The SUS score interpretation is useful, but a visual summary in a table or figure would enhance accessibility.
- **Lines 408–420:** Table 4 is helpful, though consider shading or bolding peak values to guide the reader's eye more easily.
- **Lines 499–507:** Completion time costs are clearly higher in cognitive tasks. This is a key finding and could be emphasized more directly in the results narrative.

**5. Discussion**

- **Line 526–567:** The section on clinical value is particularly strong. The inclusion of rich quotes improves the narrative. However, consider expanding on how the system may support rehabilitation beyond assessment.
- **Line 594–615:** The discussion of time and cost constraints is honest and relevant. Including a cost-effectiveness comparison to existing assessments would strengthen the argument for clinical feasibility.

**6. General Style and Language**

- Avoid overuse of colons and dashes. For example:
  - Line 74: Replace colon with “such as”
  - Line 376: Reword “tediousness and stress” to “potential monotony or stress triggers”
- Phrases such as “easy to use, fun, sport-like” (e.g., Line 28) appear often and begin to lose impact. Consider substituting synonyms or rephrasing.

**7. Figures and Tables**

- **Figure 1 and 3**: These are clear and aid understanding. However, they could benefit from more descriptive captions, particularly for readers who may not read the full text.
- **Tables**: Well formatted. However, in Table 2, adding a small sample value in the formula example would be helpful for unfamiliar readers.

**Summary**

This is an impressive and much-needed study introducing a novel tool to enhance clinical return-to-play decision-making. The writing is polished, and the methodology is rigorous for a proof-of-concept design. With minor refinements to terminology repetition, deeper justification for design decisions, and enhanced visual summaries, the manuscript will be a strong candidate for publication.

Recommendation: **Minor Revisions**
